# Supplementary material for: Addressing the Role of Angiogenesis in Patients with Advanced Pancreatic Neuroendocrine Tumors Treated with Everolimus: A Biological Prospective Analysis of Soluble Biomarkers and Clinical Outcomes
Source: Cancers (Basel). 2022 Sep 15;14(18):4471. doi: 10.3390/cancers14184471 (PMC9497075; doi:10.3390/cancers14184471)
Supplement: Supplementary file 1 [file cancers-14-04471-s001.zip › cancers-1864227-supplementary.pdf]

cancers

MDPI

Supplementary materials

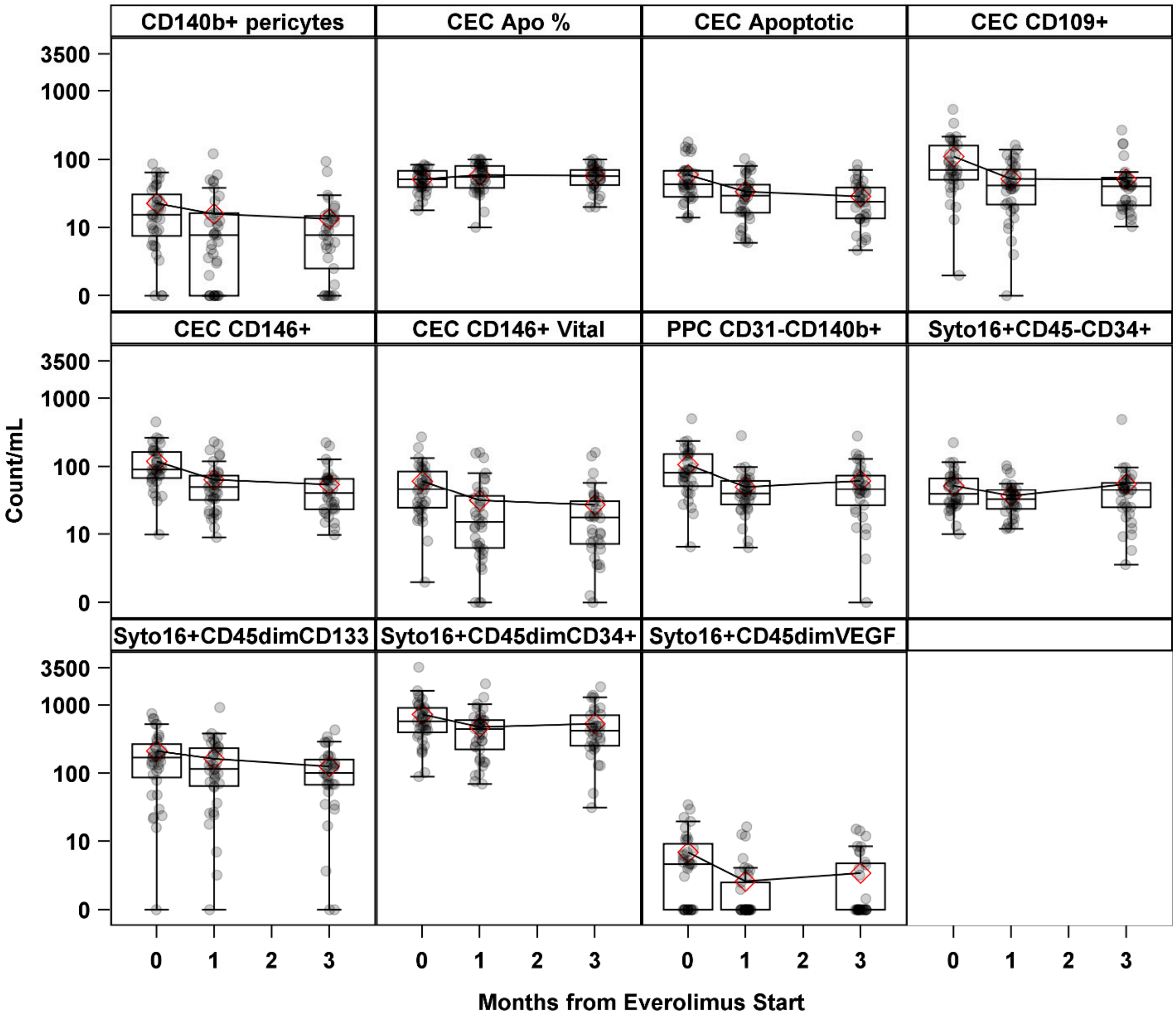

Figure S1. CCs (CECS and CEPs) by time from Everolimus start.

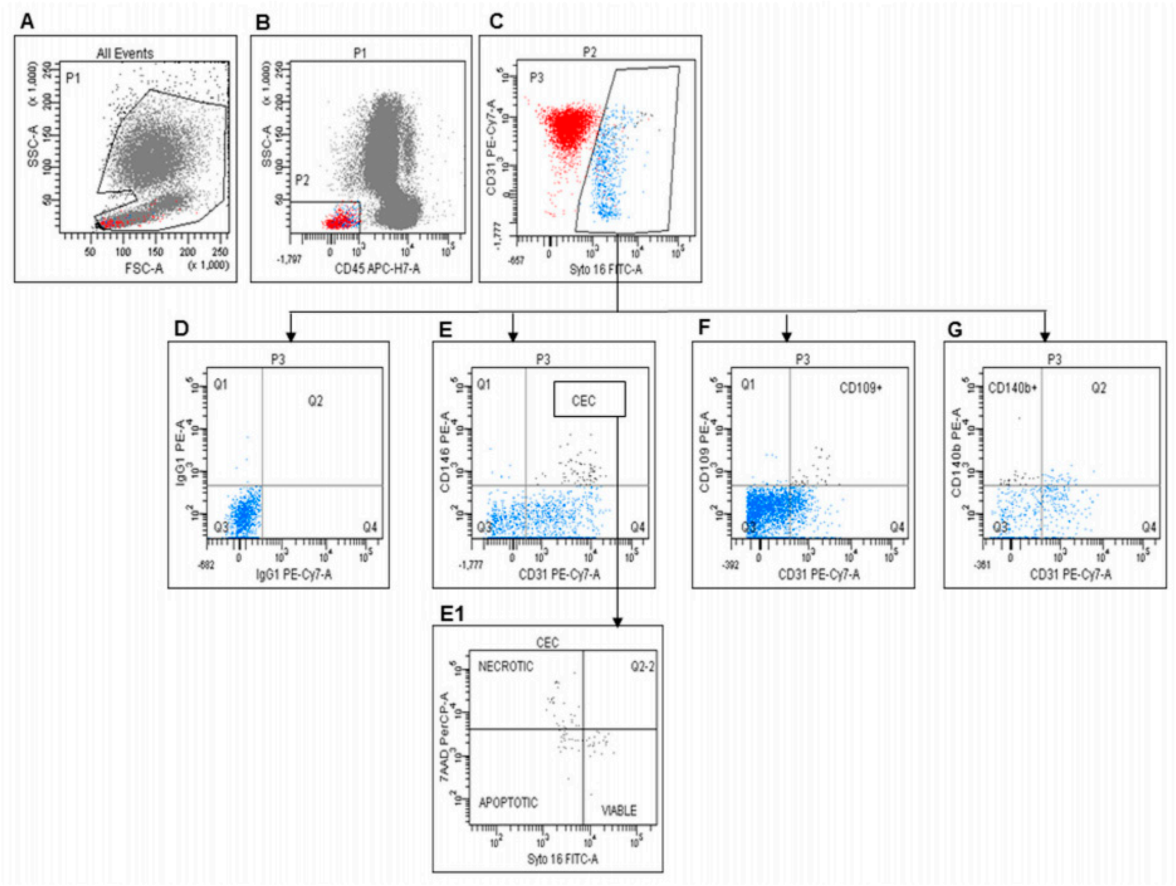

Figure S2. CCs evaluation by flow cytometry. **A**: Gate used to exclude cell fragments and debris. **B**: Gate made to identify CD45- cells. **C**: CD31 expression and Syto16 staining in CD45- cells. **D**: Negative control for **E** (CD31+ CD146+, CECs), **F** (CD31+ CD109+ CECs) and **G** (CD31+CD140b+, PPCs). **E1**: Distribution of viable, apoptotic, and necrotic CECs.

Table S1. Summary Statistics of serum BAT by time from EVE start.

	Time	N	Mean (IQR) [1]	adj p-value [2]
**TPS-1 (ng/mL)**	Baseline	38	152 (111-203)	-
	Month 1	28	142 (96-179)	0.55
	Month 3	17	166 (114-216)	0.95
	at PD	13	151 (103-169)	0.94
**VEGF (pg/mL)**	Baseline	38	448 (234-574)	-
	Month 1	28	612 (246-585)	0.02
	Month 3	17	578 (345-654)	0.09
	at PD	13	566 (356-692)	0.16
**VEGFR2 (pg/mL)**	Baseline	38	1689 (1418-1917)	-
	Month 1	28	1279 (1095-1440)	< .001
	Month 3	17	1247 (1007-1374)	< .001
	at PD	13	1583 (1375-1766)	0.05
**bFGF (pg/mL)**	Baseline	38	4.3 (1.5-6.4)	-
	Month 1	28	4.1 (1.7-5.0)	1.00
	Month 3	17	4.8 (1.7-6.5)	0.96
	at PD	13	4.0 (2.0-4.8)	0.97

[1] IQR = Inter Quartile Range; [2] Repeated Measures Adjusted p-values for Multiple comparisons vs Baseline.

Table S2. Progression Free Survival according to the first (Q1), the median and the third quartile (Q3) of the CCs at baseline.

			Cut-off value	No. Failures /At risk	Hazard Ratio (95% CI)	adj p-value [1]
**CEC CD146**	Q1	>	67.7	15/29	Ref	
		≤	67.7	6/9	1.83 (0.71-4.74)	0.21
	Median	>	90.9	10/20	Ref	
		≤	90.9	11/18	1.42 (0.60-3.35)	0.42
	Q3	>	163	4/11	Ref	
		≤	163	17/27	2.57 (0.86-7.66)	0.09
**CEC Apo (%)**	Q1	>	39.0	2/9	Ref	
		≤	39.0	19/29	3.30 (0.78-14.2)	0.11
	Median	>	51.0	8/18	Ref	
		≤	51.0	13/20	1.41 (0.58-3.40)	0.45
	Q3	>	68.0	14/27	Ref	
		≤	68.0	7/11	1.11 (0.45-2.76)	0.82
**CEC CD146 Vital**	Q1	>	24.5	16/29	Ref	
		≤	24.5	5/9	1.13 (0.41-3.09)	0.81
	Median	>	46.5	11/18	Ref	
		≤	46.5	10/20	1.24 (0.53-2.93)	0.62
	Q3	>	84.3	17/27	Ref	
		≤	84.3	4/11	1.11 (0.45-2.76)	0.82
**Apoptotic CEC**	Q1	>	24.5	15/28	Ref	
		≤	24.5	5/9	1.11 (0.40-3.05)	0.84
	Median	>	43.0	9/19	Ref	
		≤	43.0	11/18	1.77 (0.50-3.82)	0.53
	Q3	>	68.0	5/11	Ref	
		≤	68.0	15/26	1.39 (0.45-2.76)	0.82
**CD140b+ pericytes**	Q1	>	7.6	17/29	Ref	
		≤	7.6	4/9	0.73 (0.25-2.18)	0.57
	Median	>	15.5	10/19	Ref	
		≤	15.5	11/19	1.09 (0.46-2.56)	0.85
	Q3	>	30.8	4/11	Ref	
		≤	30.8	17/27	2.24 (0.75-6.69)	0.15
**CEC CD109+**	Q1	>	50.4	15/29	Ref	
		≤	50.4	6/9	1.57 (0.61-4.07)	0.35
	Median	>	69.8	11/21	Ref	
		≤	69.8	10/17	1.19 (0.50-2.79)	0.70
	Q3	>	160	5/12	Ref	
		≤	160	16/26	1.58 (0.58-4.32)	0.37
**PPC CD31 + CD140b+**	Q1	>	51.4	13/29	Ref	
		≤	51.4	8/9	3.78 (1.53-9.33)	0.01
	Median	>	81.0	10/20	Ref	
		≤	81.0	11/18	1.50 (0.64-3.54)	0.35
	Q3	>	153	7/11	Ref	
		≤	153	14/27	0.97 (0.39-2.41)	0.95
**Syto16+CD45dimCD34+**	Q1	>	401	15/29	Ref	
		≤	401	6/9	1.76 (0.68-4.56)	0.25
	Median	>	582	10/20	Ref	
		≤	582	11/18	1.73 (0.73-4.09)	0.21
	Q3	>	909	5/11	Ref	

		≤	909	16/27	1.56 (0.57-4.28)	0.39
**Syto16+CD45-CD34+**	Q1	>	27.8	17/29	Ref	
		≤	27.8	4/9	0.75 (0.25-2.23)	0.60
	Median	>	39.0	13/20	Ref	
		≤	39.0	8/18	0.58 (0.24-1.41)	0.23
	Q3	>	67.2	7/11	Ref	
		≤	67.2	14/27	0.76 (0.31-1.88)	0.55
**Syto16+CD45dimCD133+CD34+**	Q1	>	87.4	14/29	Ref	
		≤	87.4	7/9	2.70 (1.07-6.79)	0.06
	Median	>	171	10/20	Ref	
		≤	171	11/18	1.70 (0.72-4.01)	0.23
	Q3	>	269	4/11	Ref	
		≤	269	17/27	2.38 (0.80-7.09)	0.12
**Syto16+CD45dimVEGFR2+**	Q1	>	0.0	11/24	Ref	
		=	0.0	10/14	1.70 (0.72-4.02)	0.23
	Median	>	4.7	10/19	Ref	
		≤	4.7	11/19	1.21 (0.52-2.87)	0.65
	Q3	>	9.3	5/11	Ref	
		≤	9.3	16/27	1.57 (0.58-4.29)	0.38

[1] Family-wise Error Rate for multiple comparisons adjusted p-value.
